# Predicting influenza-like illness-related emergency department visits by modelling spatio-temporal syndromic surveillance data

**DOI:** 10.1017/S0950268819001948

**Published:** 2019-12-02

**Authors:** L. J. Martin, H. Dong, Q. Liu, J. Talbot, W. Qiu, Y. Yasui

**Affiliations:** 1 School of Public Health, University of Alberta, Edmonton, AB, Canada; 2 Department of Epidemiology and Cancer Control, St. Jude Children's Research Hospital, Memphis, TN, USA

**Keywords:** Emergency medical services, epidemiologic methods, population surveillance, prediction modelling, respiratory tract infections

## Abstract

Predicting the magnitude of the annual seasonal peak in influenza-like illness (ILI)-related emergency department (ED) visit volumes can inform the decision to open influenza care clinics (ICCs), which can mitigate pressure at the ED. Using ILI-related ED visit data from the Alberta Real Time Syndromic Surveillance Net for Edmonton, Alberta, Canada, we developed (training data, 1 August 2004–31 July 2008) and tested (testing data, 1 August 2008–19 February 2014) spatio-temporal statistical prediction models of daily ILI-related ED visits to estimate high visit volumes 3 days in advance. Our Main Model, based on a generalised linear mixed model with random intercept, incorporated prediction residuals over 14 days and captured increases in observed volume ahead of peaks. During seasonal influenza periods, our Main Model predicted volumes within ±30% of observed volumes for 67%–82% of high-volume days and within 0.3%–21% of observed seasonal peak volumes. Model predictions were not as successful during the 2009 H1N1 pandemic. Our model can provide early warning of increases in ILI-related ED visit volumes during seasonal influenza periods of differing intensities. These predictions may be used to support public health decisions, such as if and when to open ICCs, during seasonal influenza epidemics.

## Introduction

Influenza and influenza-like illness (ILI) can create considerable annual burdens on the healthcare system, including increases in emergency department (ED) visit volumes and hospital admissions [1, 2]. Annual epidemics of seasonal influenza, increases in other respiratory virus infections, such as respiratory syncytial virus (RSV), and variation in ILI-related ED visit volumes over the influenza season are expected. Furthermore, the timing of the highest seasonal peak in ILI-related ED visit volumes is, in general, predictable; for example, during non-pandemic years, ILI-related ED visits in Edmonton, Alberta, Canada always peaked during the Christmas-New Year holidays [3]. However, despite this predictable timing, forecasting the magnitude of the seasonal peak and the timing of high-volume days outside this peak may help to inform healthcare management decisions regarding resource allocation and staffing, and public health decisions, such as if and when to open influenza assessment centres or influenza care clinics (ICCs) to manage surges in ED visit volumes. During the 2013–2014 influenza season in Edmonton, EDs experienced especially high ILI-related visit volumes [3], prompting the opening of an ICC in January 2014 [4]. In Ontario, during the 2009 H1N1 pandemic, decisions to open influenza assessment centres were supported by evidence from syndromic surveillance systems [5]. These systems use pre-diagnostic indicators to monitor disease incidence to enable early warning of outbreaks [6].

In this study, together with the Provincial Chief Medical Officer of Health, we used daily ILI-related ED visit data from the Alberta Real-Time Syndromic Surveillance Net (ARTSSN) [7] to develop and compare spatio-temporal statistical prediction models of daily ILI-related ED visit volumes at the city level in Edmonton. Our goal was to create a model that estimated high ED visit volumes ahead of time, was easily interpretable by and useful for public health officials and ED management who already use syndromic surveillance data, and generalisable to EDs elsewhere that use similar systems.

## Methods

We accessed data from ARTSSN describing ED visits made from 1 August 2004 to 19 February 2014. ARTSSN monitors several data sources, including ED visits, telehealth calls [8] and elementary school absenteeism, in real-time [7]. Using daily ILI-related ED visit data, our goal was to predict daily ILI-related ED visit volumes 3 days ahead of time because this was the estimated amount of time required to prepare for the opening of an ICC. We defined 3 days ahead as 3 full days; for example, we used the observed data until the end of the day on 1st May to allow prediction on 2nd May for 5th May.

### Time periods

We divided our dataset into two parts: a training set for model development and validation set for model testing, similar to methods described by Harrell [9]. We used the first 4 years of data (1 August 2004–31 July 2008; 1461 days) as our training period and the remaining 5.5 years of data (1 August 2008–19 February 2014; 2029 days) as our validation period.

For years not affected by the 2009 H1N1 pandemic, we analysed data collected from 1 August of one calendar year to 31 July of the following calendar year (2004–2008 and 2010–2013) to incorporate an entire influenza season; however, the data for the 2013–2014 season ended on 19 February 2014. For pandemic-affected years, we divided time periods to account for and examine the predictive abilities of our models during the 2009 H1N1 pandemic. To this end, we defined the pre-H1N1 period as 1 August 2008–31 March 2009; the first wave of H1N1 as 1 April 2009–31 July 2009; the second wave of H1N1 as 1 October 2009–5 December 2009 and the post-H1N1 period as 6 December 2009–31 July 2010. Thus, we have an extended 2009–2010 season from 1 April 2009–31 July 2010 to account for the 2-month period between the first and second H1N1 waves. The definitions of the pandemic wave periods correspond approximately to those described in Alberta's report on the pandemic [10].

### Healthcare use and population data

We used ED visit data from nine Edmonton-area hospitals, three of which began providing data after the start of the study period: two during our training period in 2004 and 2005 and one during our testing period in 2013 (which added approximately *n* = 88 visits). For each ED visitor, we included the following variables: visit date, date of birth, forward sortation area (FSA) of the postal code and chief complaint. We grouped patient age into seven categories (⩽1 year and 2–4, 5–8, 9–17, 18–64, 65–74 and ≥75 years). We defined ILI-related visits as those with a chief complaint related to ‘cough’. In our previous work using these data, this definition was significantly associated with laboratory detections for both influenza A and RSV and had higher correlations with these detections compared to the chief complaint ‘fever’ [3]. However, the chief complaint ‘cough’ has the potential to both include individuals with non-ILI-related illnesses as well as exclude individuals with ILI who do not present with a cough. In these ED data, only one chief complaint can be entered for each visit; therefore, it is not possible to test combinations of symptoms, such as fever and cough, which are commonly used to define ILI. We limited the data to ILI-related ED visits for Edmonton residents (2006 population = 733 970 [11]; this excluded approximately 37% of the total ILI-related visits made to the hospitals in our analysis), based on their FSA (*n* = 36 FSAs), and divided the city into five areas (northeast; downtown; west; southeast and southwest). We included all eligible visits, regardless of the discharge disposition (e.g. we included visits from patients who had left without being seen). In addition to data from ARTSSN, we used the 2006 census of the population to calculate population rates by FSA and age [11]. We received approval from the University of Alberta Health Research Ethics Board to access and analyse ARTSSN data.

### Model development

Our Main Model consisted of three elements: (1) generalised linear mixed model (GLMM) (without spatio-temporal characteristics), (2) spatio-temporal characteristics and (3) an indicator variable to capture increases in volume at the beginning of peaks. In comparison, we examined two reduced versions of our Main Model, which we refer to as Models 2 and 3, and a simple non-parametric approach that used the count 3 days ahead directly as the predicted count for the day of interest. Comparing these subsets of the Main Model allowed us to see how each element of the modelling contributed to the prediction capabilities.

### Main Model

#### Generalised linear mixed model with a random intercept

Our Main Model was based on a Poisson regression model with daily ILI-related ED visits *P*-days ahead of the day of interest as the outcome, where *P* is the number of days ahead for which we wished to make a prediction (we set *P* = 3), and log transformed area- and age-specific population as the offset. As predictors, we included the following main effects: (1) age group; (2) area of the city; (3) Christmas and New Year holiday period (24 December–3 January); (4) day of the week and (5) natural cubic splines to represent the day of the year (*X* = 1, 2, …, 365 or, for a leap year, *X* = 1, 2, …, 366). We explored various values for the number of knots to be used in the natural cubic splines and chose the final value of 15 based on the Akaike Information Criterion [12] of model with the main effects. We *a priori* hypothesised seven possible interactions: (1) age group and day of the year; (2) age group and area; (3) age group and day of the week; (4) age group and holiday; (5) area and day of the week; (6) area and day of the year and (7) area and holiday. We used forward selection with the significance level for entry as 0.05, which resulted in three statistically significant two-way interactions: age and day of the year, age and area, and age and day of the week. Using GLMM, we added a random intercept following a Gaussian distribution to the model to capture the extra Poisson day-to-day variation; thus, over-dispersion is included in the GLMM, which is described below in Equation (1):

Systematic part:
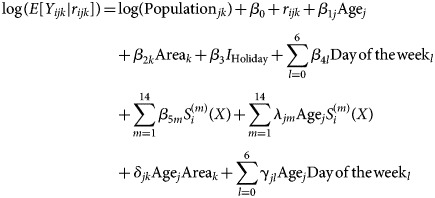


Random part:
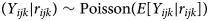

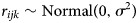


Specifically, let *Y*_*ijk*,_ *i* = 1, 2, …, 1461; *j* = 1, 2, …, 7; *k* = 1, 2, …, 5 be the daily ILI-related ED visit count on the *i*th day in *j*th age group and the *k*th area; *S*^(*m*)^ (*i*), *m* = 1, 2, …, 14 be the explanatory variables for the systematic seasonal variation represented by the natural cubic splines; and *r*_*ijk*_ be the random day-to-day extra-Poisson Gaussian variation. To capture the seasonal variation by *S*^(*m*)^ (*i*), *m* = 1, 2, …, 14, we placed the first knot at Day 15 (15th January) and the last knot at Day 350 and placed the middle 13 knots at equal distances in between (i.e. we divided the interval from Day 15 to Day 350 into equal pieces). The GLMM provides the first component of our predicted ILI-related ED visit count from its systematic part.

#### Spatio-temporal characteristics

To construct the second component of our predicted ILI-related ED visit count, we calculated the daily residual *R*_*ijk*_ as the difference between the observed and the GLMM-predicted ILI-related ED visit counts for each of the age group and area combinations. Then, we summarised this residual information over a *C*-day window that is 3 days ahead of the predicted day using a mean residual: 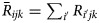 where the summation is over *i*^′^ = *i* − *P* − *C*,  *i* − *P* − *C* + 1, …, *i* − *P* − 1, and *P* = 3 days ahead of the predicted day. To capture the potential relationship between ED visits among neighbourhoods, we incorporated the residual information from the four areas other than the area under prediction. To do this, we fit a linear regression model with *R*_*ijk*_ being the outcome and using 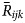 and the mean, median and maximum of the 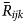 for the other four areas as four predictors to calculate the predicted residual 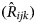. This locally predicted residual is the second component of our predicted ILI-related ED visit count which attempts to capture local spatio-temporal variations over and beyond the GLMM-predicted means and gives more weight to recent data.

We considered four possible values of *C* (3, 7, 14 and 21) and chose 14 based on cross-validation of predicting the observed ILI-related ED visit count of ≥25 or <25 visits using data from 1 August 2004 to 31 July 2009 (the training data, pre-H1N1 period and the first wave of the H1N1 pandemic); 25 visits was the 75th percentile of daily counts of ILI-related visits in this portion of the data.

#### Capturing the beginning of a peak in the ILI-related ED visit count

The last component of our predicted ILI-related ED visit count was motivated by our observation that the sum of the two components of our predicted ILI-related ED visit count (i.e. the GLMM-predicted means + the locally predicted residual) was not sensitive enough to sudden changes in the observed ILI-related ED visit count at the beginning of its peak. That is, these sums were often lower (i.e. underpredicted) than the observed counts. To improve the prediction for peak periods, which are the critical periods of interest, we created a ‘hockey stick-like term’ (HSLT) described below, similar to a hinge function [13], and included it as the third, final component of our predicted ILI-related ED visit count.

Specifically, we utilise 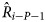, the residual of the day that is *P*-days ahead (the last day in the 14-day time window, closest to the 3 days that are the prediction target) and let it inform us whether the peak might be coming. That is, we create 
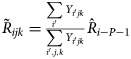
 as the fraction of 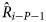 distributed to the *j*^th^ age group and *k*^th^ area. Then we let 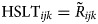 if 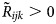 and HSLT_*ijk*_ = 0 otherwise: this function takes a shape similar to an ice-hockey stick. Adding this HSLT to the sum of the GLMM-predicted means plus the locally predicted residuals completes our predicted ILI-related ED visit count (thereafter referred to as ‘Main Model’) and allows it to be more sensitive to the most recent time trend, increasing at the beginning of a peak. The following equation (Equation (2)) describes the locally predicted residuals and the HSLT:

Systematic part:
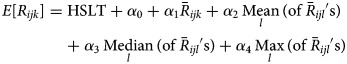


Random part:
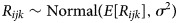
where *i* = 1, 2, …, 1461, *j* = 1, 2, …, 7, *k* = 1, 2, …, 5, *l* = 1, 2, …, 5, *l* ≠ *k* (where *l* represents the areas other than *k*).

The final estimated count for our Main Model is the sum of the estimates obtained from Equation (1) plus Equation (2).

### Models for comparison

To examine the performance of the three components of our predicted ILI-related ED visit count independently, we assessed two reduced models: Model 2 included only the GLMM-based mean component (i.e. we excluded both the locally-predicted residual and HSLT) while Model 3 included only the GLMM-based mean component and the locally predicted residual but without the HSLT. For our final approach for comparison, we employed a simple non-parametric method that used the count 3 days ahead directly as the predicted count for day of interest.

### Model evaluation

We tested and compared the performance of our models and the non-parametric approach above using the testing data. As we assumed predicting days with high rather than low visit volumes would be of primary interest, we evaluated our models based on their ability to predict higher volume days. We defined a high-volume day as one in which the number of ILI-related ED visits exceeded the 95th percentile of the training data (2004–2008). In addition to defining peak high-volume days, we examined the maximum seasonal peak volume and assessed how closely the observed and predicted seasonal peaks were in terms of magnitude and timing for our Main Model, which is of interest retrospectively. We evaluated and compared all models based on their ability to predict peak days and examined how closely our predictions estimated observed values. Our main evaluation criterion was based on what we considered to be of importance to healthcare system planning: the percentage of days in which the predicted volume was within ±30% of the observed volume, focusing again on high-volume days. In addition, we examined how well the model estimated the highest peak volumes each period and calculated area under the receiver operator characteristic curve (AUC), root mean squared error (RMSE) and the relative percentage difference between the observed and estimated visit volumes for each model over each period and for each Christmas-New Year holiday. To visualise model performance, we also examined plots comparing predicted visit volumes for each model to observed visit volumes. We analysed the data using R (versions 2.14.2 and higher) [14].

## Results

### ILI-related ED visit volumes

The median visit volumes in our training and validation datasets differed. During the 1461-day training period (2004–2008), the median visit volume was 17 visits/day (range by season = 16–19 visits/day) and the 95th percentile was 36 visits/day; we used this value to define a high-volume day as one with a visit volume ≥36 visits/day. In comparison, during the 2029-day testing period (2008–2014), visit volumes were, overall, higher (median of 23 visits/day and 95th percentile of 49 visits/day) and the median daily visit volumes varied more from season-to-season than during the training period (Table 1). During the extended 2009–2010 season (1 April 2009–31 July 2010), the median visit volume was 22 visits/day; however, median visit volumes were much higher during the first and second pandemic H1N1 waves (25 visits/day and 50 visits/day, respectively; Table 1). Over the last four seasons of the testing period (2010–2014), the median visit volumes increased from 21 visits/day in 2010–2011 to 22.5 visits/day in 2011–2012, 26 visits/day in 2012–2013 and 32 visits/day in the first part of 2013–2014 (Table 1).

**Table 1.** Comparing observed *vs*. predicted maximum peaks in daily ILI-related ED visit volumes, in terms of magnitude and timing, Edmonton, Alberta, 2008–2014

Time period	Median no. of visits/day	Observed peak ILI-related visit volume and date		Predicted^a^ peak ILI-related visit volume and date		Difference between observed and predicted^a^ maximum peak ILI-related volumes (relative %) and date		No. (%) of the 7^b^ days ahead of the seasonal peak when predicted volume was within 30% of observed volume			
								Non-parametric method	Model 2: GLMM	Model 3: GLMM and locally-predicted residual	Main Model
Pre-H1N1 (1 Aug 08–31 Mar 09)	19	52 47	Thu, Jan 1/09 Mon, Mar 2/09	42.5 47.6	Sun, Dec 28/08 Sun, Feb 22/09	−9.5 (−18.2%) 0.6 (1.2%)	−4 days −8 days	9 (64.3)	9 (64.3)	11 (78.6)	11 (78.6)
H1N1 Wave 1 (1 Apr 09–31 Jul 09)	25	57	Sun, May 3/09	58.9	Mon, May 4/09	1.9 (3.3%)	+1 day	5 (71.4)	1 (14.3)	2 (28.6)	3 (42.9)
H1N1 Wave 2 (1 Oct 09 –5 Dec 09)	50	275	Wed, Oct 28/09	297.3	Sun, Nov 1/09	22.3 (8.1%)	+4 days	2 (28.6)	0 (0.0)	0 (0.0)	3 (42.9)
Post-H1N1 (6 Dec 09–31 Jul 10)	20.5	51	Tue, Mar 2/10	50.2	Sat, Mar 6/10	−0.8 (−1.6%)	+4 days	3 (42.9)	7 (100)	5 (71.4)	5 (71.4)
2010–11 (1 Aug 10–31 Jul 11)	21	62	Sun, Dec 26/10	48.7	Sun, Dec 26/10	−13.3 (−21.4%)	0 days	2 (28.6)	5 (71.4)	5 (71.4)	4 (57.1)
2011–12 (1 Aug 11–31 Jul 12)	22.5	59 54	Sun, Jan 1/12 Tue, Feb 28/12	50.5 53.9	Mon, Dec 26/11 Sat, Mar 3/12	−8.5 (−14.4%) −0.1 (−0.3%)	−6 days +4 days	7 (50.0)	14 (100)	14 (100)	12 (85.7)
2012–13 (1 Aug 12–31 Jul 13)	26	125	Wed, Dec 26/12	145.6	Sun, Dec 30/12	20.6 (16.5%)	+4 days	6 (85.7)	0 (0.0)	1 (14.3)	7 (100)
2013–14 (1 Aug 13–15 Feb 14)	32	93	Sun, Dec 29/13	110.3	Sun, Dec 29/13	17.3 (18.6%)	0 days	4 (57.1)	0 (0.0)	1 (14.3)	6 (85.7)

a Predicted volumes are based on our Main Model.

b The pre-H1N1 and 2011–2012 season each had two similarly sized maximum peak volumes that occurred at notably distinct times within each of these periods; therefore, we examined the combined no. (%) over the 7 days ahead of each of these peaks (14 days total).

### Model evaluation

#### Visual comparisons

**Fig. 1.**
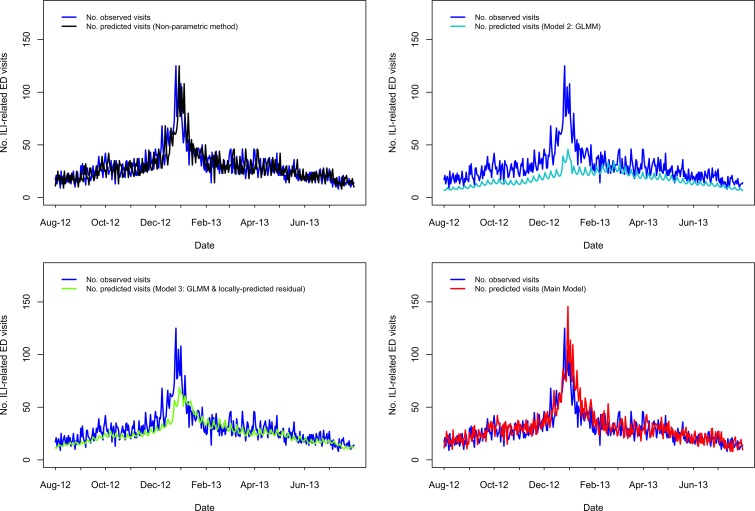
Comparing the predicted and observed number of ILI-related ED visits for each method for the 2012–2013 influenza season (1 August 2012–31 July 2013), Edmonton, Alberta. Observed visit volumes (blue) are compared to predicted visit volumes from the non-parametric method (black), Models 2 (light blue) and 3 (green) and the Main Model (red).

**Fig. 2.**
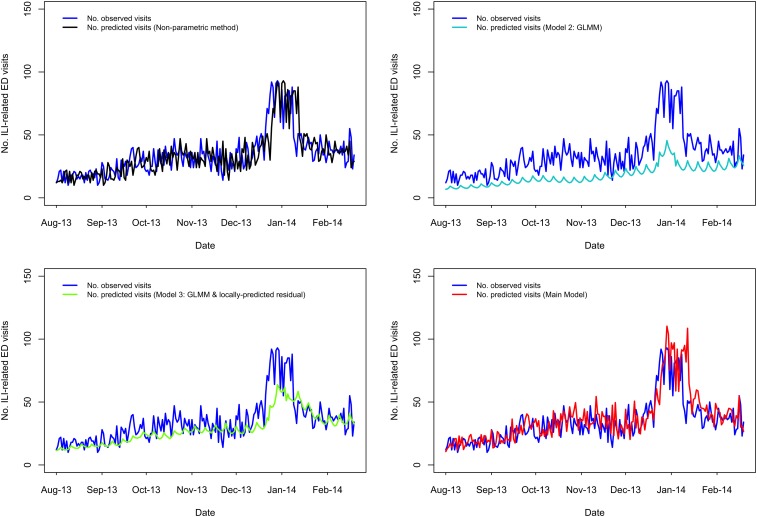
Comparing the predicted and observed number of ILI-related ED visits for each method for the 2013–14 influenza season (1 August 2013–19 February 2014), Edmonton, Alberta. Observed visit volumes (blue) are compared to predicted visit volumes from the non-parametric method (black), Models 2 (light blue) and 3 (green) and the Main Model (red).

**Fig. 3.**
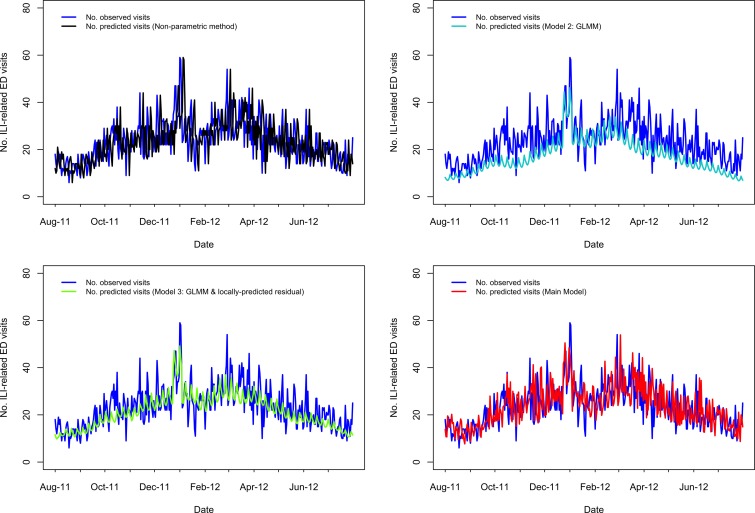
Comparing the predicted and observed number of ILI-related ED visits for each method for the 2011–2012 influenza season (1 August 2011–31 July 2012), Edmonton, Alberta. Observed visit volumes (blue) are compared to predicted visit volumes from the non-parametric method (black), Models 2 (light blue) and 3 (green) and the Main Model (red).

**Fig. 4.**
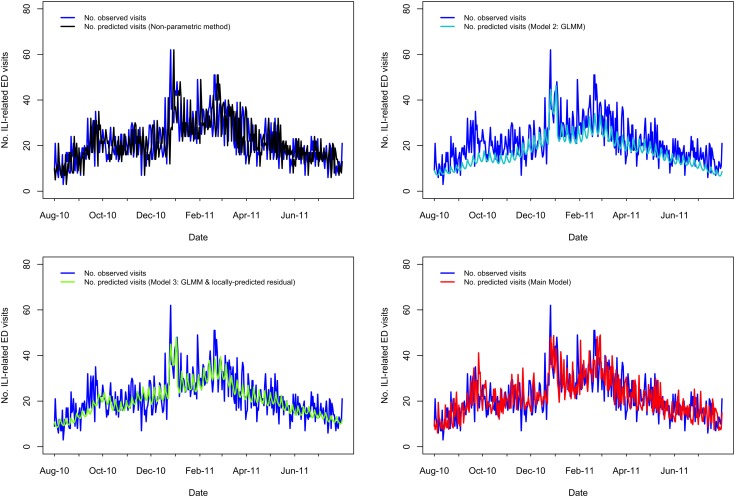
Comparing the predicted and observed number of v-related ED visits for each method for the 2010–11 influenza season (1 August 2010–31 July 2011), Edmonton, Alberta. Observed visit volumes (blue) are compared to predicted visit volumes from the non-parametric method (black), Models 2 (light blue) and 3 (green) and the Main Model (red).

A visual comparison of predicted *vs.* observed volumes for each model illustrates the differences in the models' abilities to capture variation in the visit volumes over time (Fig. 1–4; Supplementary Figs S1–S4). The Main Model and the non-parametric model responded to variation in observed volumes most effectively; this is especially obvious in the high-volume seasons, 2012–2013 and 2013–2014 (Figs 1–2). Figure 2 (Main Model) appears to show evidence of the ICC that opened in January 2014. That is, the predicted volumes from our Main Model were much higher than the observed volumes during the period in mid-January that the ICC was opened; note that visits to the ICC were not incorporated into observed data.

#### Area under the curve, RMSE and relative percentage difference

Defining a high-volume day as one with ≥36 visits, AUC values ranged from 0.600 to 0.969 considering all models (Supplementary Table S1). AUC values for the Main Model ranged from 0.710 (H1N1 wave 1) to 0.921 (H1N1 wave 2) over all periods and, outside the H1N1 period, ranged from 0.818 to 0.896 (Supplementary Table S1). We provide RMSE in Supplementary Table S2 and the relative percentage difference between observed and estimated visit volumes in Supplementary Figures S5 and S6.

#### Predicting maximum seasonal peak visit volumes

Our Main Model predicted the timing of seasonal peak visit volumes to occur between 8 days before and 4 days after the date of observed seasonal peaks and frequently predicted seasonal peaks to occur on Sundays (Table 1). Predicted peaks for the first and second waves of the 2009 H1N1 pandemic were 4 May 2009 and 1 November 2009, whereas the observed peaks were 3 May 2009 and 28 October 2009, respectively. However, even though the predicted peak for the first pandemic wave was only 1 day later than the observed peak, this prediction was responding to the increase that had already been observed in the first part of the peak, rather than predicting the highest volume that followed (Supplementary Fig. S2). Using the Main Model, over all the time periods examined in the testing data, the magnitude of the predicted peak visit volume was between 13.3 visits lower and 22.3 visits higher than the observed peak volume, or a relative percentage difference from the observed peak magnitude of between 0.3% and 21.4% (Table 1).

#### Seven days ahead of maximum seasonal peaks

Outside of the 2009 H1N1 pandemic waves, in the 1- to 7-day period immediately preceding the highest peak(s), the percentage of days in which the predicted volume was within 30% of the observed volume was highest for our Main Model (ranging from 57% to 100%), while ranging from 29% to 86% for the non-parametric method; 0% to 100% for Model 2; 14% to 100% for Model 3 (Table 1). Aside from the 2009 H1N1 pandemic, the two influenza seasons during our study period with the highest visit volumes were 2012–2013 followed by 2013–2014. In 2012–2013, predictions from our Main Model were within 30% of observed volumes for 100% of the 7 days leading up to the seasonal peak, which occurred Wednesday, 26 December 2012 (Table 1; Fig. 1); however, our Main Model predicted that the highest peak volume would occur 4 days later, on Sunday, 30 December 2012 (Table 1). In 2013–2014, our Main Model predicted the correct date of the peak (Sunday, 29 December 2013), with a predicted magnitude approximately 17 visits greater than observed (Table 1; Fig. 2). By comparing our Main Model to Models 2 and 3 (both GLMMs), we see that each of these models also predicted the peak would occur 29 December 2013 (Fig. 2). Therefore, the peak predicted by our Main Model was not due to temporally local changes in visit volumes, such as the almost identical peak that occurred previously (i.e. on 25 December 2013 the visit volume rose to *n* = 92 visits; Fig. 2), but to the systematic variations in visit volumes.

#### Percentage of high-volume days with predicted visit volumes within 30% of observed visit volumes

Of the 1542 days occurring outside the extended 2009–2010 season (i.e. excluding the 1 April 2009–31 July 2010 period), between 8.5% (2010–2011, *n* = 31 days) and 39% (2013–2014, *n* = 79 days) were high-volume days (Table 2). Considering all models, predicted visit volumes were within 30% of the observed visit volumes for between 2.5% (*n* = 2 days, Model 2, 2013–2014) and 86% (*n* = 19 days, Model 3, pre-H1N1 period) of these high-volume days outside the extended 2009–2010 season (Table 2). Our Main Model was consistent, with between 67% (2011–2012, *n* = 22 days) and 82% (pre-H1N1, *n* = 18 days) of high-volume days having predicted volumes within 30% of the observed volumes, and achieved the highest percentage during three of these five time periods outside the extended 2009–2010 season and also the highest percentage (71%, *n* = 12 days) during the post H1N1 period (Table 2). Using this metric, Model 2 (GLMM) did not perform as well as the other models during the higher volume seasons (2012–2013 and 2013–2014) (Table 2).

**Table 2.** Number and percentage of days in which the predicted visit volume was within 30% of the observed visit volume, Edmonton, Alberta, 2008–2014

Model	Pre-H1N1 (1 Aug 2008–31 Mar 2009)	Extended 2009–2010 season				2010-2011 (1 Aug 2010–31 Jul 2011)	2011–2012 (1 Aug 2011–31 Jul 2012)	2012–2013 (1 Aug 2012–31 Jul 2013)	2013–2014 (1 Aug 2013–19 Feb 2014)
		Whole period (1 Apr 2009–31 Jul 2010)	H1N1 Wave 1 (1 Apr 2009–31 Jul 2009)	H1N1 Wave 2 (1 Oct 2009–5 Dec 2009)	Post-H1N1 (6 Dec 2009–31 Jul 2010)				
All days included in calculation									
Total no. of days in period	243	487	122	66	238	365	366	365	203
Main Model	168 (69.1%)	285 (58.5%)	66 (54.1%)	29 (43.9%)	156 (65.5%)	235 (64.4%)	253 (69.1%)	238 (65.2%)	131 (64.5%)
Model 2: GLMM	178 (73.3%)	227 (46.6%)	27 (22.1%)	12 (18.2%)	160 (67.2%)	227 (62.2%)	194 (53.0%)	105 (28.8%)	31 (15.3%)
Model 3: GLMM and locally-predicted residual	**186 (76.5%)**	**319 (65.5%)**	**87 (71.3%)**	18 (27.3%)	**167 (70.2%)**	**269 (73.7%)**	**298 (81.4%)**	**272 (74.5%)**	**144 (70.9%)**
Non-parametric method	149 (61.3%)	268 (55.0%)	65 (53.3%)	**34 (51.5%)**	131 (55.0%)	199 (54.5%)	211 (57.7%)	230 (63.0%)	127 (62.6%)
Only high volume (≥36 visits) days included in calculation									
No. of days in each period with ≥36 visits (% of total days in period)	22 (9.1)	71 (14.6)	15 (12.3)	39 (59.0)	17 (7.1)	31 (8.5)	33 (9.0)	84 (23.0)	79 (38.9)
Main Model	18 (81.8%)	30 (42.3%)	**8 (53.3%)**	10 (25.6%)	**12 (70.6%)**	23 (74.2%)	**22 (66.7%)**	**58 (69.0%)**	**59 (74.7%)**
Model 2: GLMM	11 (50.0%)	8 (11.3%)	0 (0.0%)	0 (0.0%)	8 (47.1%)	17 (54.8%)	13 (39.4%)	7 (8.3%)	2 (2.5%)
Model 3: GLMM and locally-predicted residual	**19 (86.4%)**	18 (25.4%)	3 (20.0%)	5 (12.8%)	10 (58.8%)	**24 (77.4%)**	21 (63.6%)	50 (59.5%)	45 (57.0%)
Non-parametric method	13 (59.1%)	**34 (47.9%)**	**8 (53.3%)**	**17 (43.6%)**	9 (52.9%)	15 (48.4%)	13 (39.4%)	54 (64.3%)	55 (69.6%)

*Note*: The highest percentage(s) in each period is shown in bold.

Of the 188 days occurring during the first and second H1N1 waves, 54 (29%) were high-volume days: 15 days during the first wave and 39 days during the second wave (Table 2). Using the non-parametric method, predicted volumes for 53% (*n* = 8 days) of these high-volume days during the first wave and 44% (*n* = 17 days) of these high-volume days during the second wave were within 30% of the observed volume, which were the highest combined percentages during these two periods (Table 2). Using this metric, our Main Model performed similarly well as the non-parametric method during the first pandemic wave, with predicted volumes within 30% of observed volumes for the same percentage of high-volume days (53%) (Table 2). However, during the second pandemic wave, our Main Model met this criterion for only 26% (*n* = 10 days) of high-volume days. Models 2 and 3 did not perform as well compared to the other models during the H1N1 waves based on this metric (Table 2).

## Discussion

We developed and compared prediction models to forecast ILI-related ED visit volumes 3 days in advance, incorporating variation by visitor age, area of residence and season using completely separate datasets for model development and model validation. Based on our main evaluation criterion (percentage of high-volume days in which the predicted volume was within ±30% of the observed volume), our Main Model performed most effectively outside of the 2009 H1N1 pandemic period and improved upon the use of the volume of the current day directly as a prediction for the volume 3 days ahead of time.

During seasonal influenza periods, our Main Model discriminated high-volume days, demonstrated by high AUC values; predicted volumes using our Main Model were within 30% of observed volumes for 67% to 82% of high-volume days and within 0.3% and 21% of the observed maximum seasonal peak visit volumes. In comparison, for the non-parametric method that directly predicted the volume 3 days ahead of time, AUC values were somewhat lower during seasonal influenza periods and predicted volumes were within 30% of observed volumes for only 39% to 70% of high-volume days; for this method, maximum peak volumes matched exactly, but were always 3 days late.

By modelling the residuals, we were able to account for changes in volumes over time, allowing our model to respond to increases in volume beyond what would have been predicted using the training data alone. This was especially important given that higher visit volumes occurred in our validation dataset compared to our training dataset. Furthermore, by incorporating an indicator variable (HSLT) in the model, we were better able to predict increases in volume during high-volume days, which was most evident during the 2012–2013 and 2013–2014 influenza seasons. Possible alternative approaches to the use of the HSLT could be weighting residuals based on how recently they occurred relative to the day in question. A strength of our approach is that the patterns in the ILI-related ED visits predicted by our model using syndromic data are related to patterns in laboratory detections for both influenza A and RSV [3]. However, our models did not perform as well during the 2009 H1N1 pandemic period; as Viboud *et al*. suggest, compartmental models may be more useful in this type of scenario [15].

One of our goals was to create a model to inform decisions regarding the opening of an ICC. These decisions require estimation of the expected timing and intensity of the peak in ILI-related ED visit volumes. During non-pandemic seasons, the *timing* of this peak can be expected during the Christmas-New Year holidays [3], which provides an indication of when such a clinic should likely be opened. However, determining if an ICC should be opened also requires estimating the expected *intensity* of the peak volume; this estimation requires additional evidence, which our model can provide. For example, our Main Model predictions were within 30% of the observed volumes for 7 and 6 of the 7 days leading up to the date of the maximum seasonal peaks in 2012–2013 and 2013–2014, respectively, which improved upon the other methods we explored. The next best model was the non-parametric approach, for which 6 and 4 of the 7 days leading up to the date of the maximum seasonal peak in 2012–2013 and 2013–2014, respectively, were within 30% of the observed volumes. The other two models met this objective for either 0 or 1 of these 7 days. These two seasons are most important to consider because of high volume (in 2012–2013) and the decision to open an ICC (in 2013–2014). Furthermore, the volume during these 7 days is likely a critical consideration in deciding whether or not to open an ICC; therefore, earlier and more accurate estimates are especially important at this time.

Aspects of our modelling approach are similar to those of Kleinman *et al*. [16], who used GLMM with spatio-temporal data. Other than census data, our models did not rely on data external to the ED. Although others have considered telehealth data for forecasting ILI-related ED visits [17], we found that these data did not improve our model predictions (results not shown). In addition to considering telehealth data as a possible external data source for modelling ILI-related ED visits, other prediction models have incorporated weather-related data [18] and Google Flu Trends estimates [19]. Although these types of external data sources may be useful for predictions, we believe that limiting models to internal data is also advantageous. First, it is a simpler approach, as no other data are required beyond what are currently collected by the medical centres themselves and available through the census. Second, it reduces the risk of the model being impacted by changes in these external data (e.g. changes in the Google Flu Trends algorithm [20] or loss of availability of its data [21]). Finally, it makes the models more easily transferable to other real-time, electronic ED surveillance systems regardless of the availability of additional data in these systems.

This study and our models have several limitations. First, changes in the data occurred during the study that likely impacted our estimates. During both the 2009 H1N1 pandemic and the 2013–2014 influenza seasons, ILI-related ED visits decreased after the influenza assessment centres or care clinics were opened. Since our model did not consider these external visits, these decreases in volume likely impacted our models' estimates. Additionally, we did not include the total number of ED visits in our analysis, so we cannot examine surges in overall volume or ILI-related ED visits as a proportion of all visits. Second, although the population of Edmonton grew considerably over the study period, we used only the 2006 census of the population to determine rates. However, the impact of this is alleviated to some extent because we modelled the residuals rather than the counts directly. Third, we did not consider long-term trends in our modelling approach. Doing so could help to account for the increase in the number of ILI-related ED visits observed between the training and testing data and the increasing RMSE observed over time. However, we do not know how well a long-term trend would truly apply in the long term, especially given economic and political changes that have occurred in Alberta over the past several years. Fourth, Edmonton residents may have attended EDs outside the city, which would not be represented in these numbers. Fifth, we did not consider epistemic uncertainty in our analysis. Finally, we did not break down our predicted volumes by area of the city, hospital or patient age group because of the consequential reduction in model power and precision. However, most visits were made to two large EDs in the city and our model predictions could be roughly estimated for each hospital by considering the proportion of visits occurring in each area or hospital.

Our recommendations for future work include examining model refinements. For example, model predictions could be updated after the 3-day window of advanced warning; that is, update estimates post-prediction. Additionally, predictions at a smaller geographic area or at the hospital-level, and by age category could also be explored as well as other methods to model the spatial distribution of the visits across the city. Finally, model performance could be evaluated based on other influenza season characteristics, including the main influenza virus subtypes in circulation; incidence of other respiratory viruses, such as RSV; and vaccine timing and effectiveness. Furthermore, our model should be tested in practice to determine its usefulness in public health preparedness and decision-making during the influenza season.

In conclusion, we developed spatio-temporal models to predict ILI-related ED visits 3 days in advance to help inform healthcare and public-health decision-making, such as the opening of an ICC, especially during seasonal influenza periods. Based on our evaluation, the most effective modelling approach considered was our Main Model, which was based on a GLMM with random intercept and incorporated spatio-temporal characteristics as well as an indicator variable similar to a hinge function. Our approach herein provides an example of how syndromic surveillance data can be used to provide early warnings of increases in healthcare use for the monitored syndrome itself. These models can be further tested in practice and adapted to other real-time, electronic surveillance systems monitoring ILI-related ED visits, and potentially modified for other infectious diseases.

## Supplementary material

For supplementary material accompanying this paper visit https://doi.org/10.1017/S0950268819001948. click here to view supplementary material
