# Supplementary material for: Predicting influenza-like illness-related emergency department visits by modelling spatio-temporal syndromic surveillance data
Source: Epidemiol Infect. 2019 Dec 2;147:e312. doi: 10.1017/S0950268819001948 (PMC7003624; doi:10.1017/S0950268819001948)
Supplement: Supplementary file 1 [file S0950268819001948sup001.docx]

**Supplementary Materials**

**Article title:** Predicting influenza-like illness-related emergency department visits by modelling spatio-temporal syndromic surveillance data

**Authors:** Leah J Martin*, Huiru Dong, Qi Liu, James Talbot, Weiyu Qiu, Yutaka Yasui

*leahjmartin1@gmail.com, leah.martin@ualberta.ca

**Journal:** Epidemiology and Infection

**Contents:** Supplementary Tables S1-S2, Supplementary Figures S1-S6, Notes on Supplementary Figures S5 and S6

**Supplementary Table S1.** Area under the receiver operating curve (AUC) for each model, defining a high-volume day as one with ≥36 visits, by period, Edmonton, Alberta, 2008-2014

|  | **Pre-H1N1** | **H1N1 Wave 1** | **H1N1 Wave 2** | **Post-H1N1** | **Extended 2009-2010 season** | **2010-2011** | **2011-2012** | **2012-2013** | **2013-2014** |
| --- | --- | --- | --- | --- | --- | --- | --- | --- | --- |
| Non-parametric Method | 0.814 | 0.712 | **0.934** | 0.776 | 0.881 | 0.782 | 0.699 | 0.846 | 0.798 |
| Model 2: GLMM | 0.925 | **0.750** | 0.719 | 0.829 | 0.600 | **0.969** | 0.867 | 0.893 | 0.857 |
| Model 3: GLMM and locally-predicted residual | **0.933** | 0.683 | 0.784 | **0.873** | 0.859 | 0.966 | **0.879** | **0.932** | **0.861** |
| Main Model | 0.888 | 0.710 | 0.921 | 0.834 | **0.894** | 0.896 | 0.818 | 0.890 | 0.837 |

*Note:*The model with the largest AUC for each period is in bold.

**Supplementary Table S2.** Root mean squared error (RMSE) for each method, by period, Edmonton, Alberta, 2008-2014

|  | **Pre-H1N1** | **H1N1 Wave 1** | **H1N1 Wave 2** | **Post-H1N1** | **Extended 2009-2010 season** | **2010-2011** | **2011-2012** | **2012-2013** | **2013-2014** |
| --- | --- | --- | --- | --- | --- | --- | --- | --- | --- |
| Non-parametric method | 8.19 | 9.90 | 40.78 | 8.63 | 17.04 | 9.00 | 9.29 | 10.99 | 12.23 |
| Model 2: GLMM | 6.16 | 14.05 | 85.35 | 7.31 | 32.68 | 7.22 | 8.51 | 16.70 | 20.57 |
| Model 3: GLMM and locally-predicted residual | 5.65 | 9.06 | 61.33 | 6.08 | 23.47 | 5.70 | 5.98 | 10.01 | 11.94 |
| Main Model | 6.63 | 9.52 | 46.90 | 7.19 | 18.70 | 7.09 | 7.55 | 10.50 | 12.47 |

**
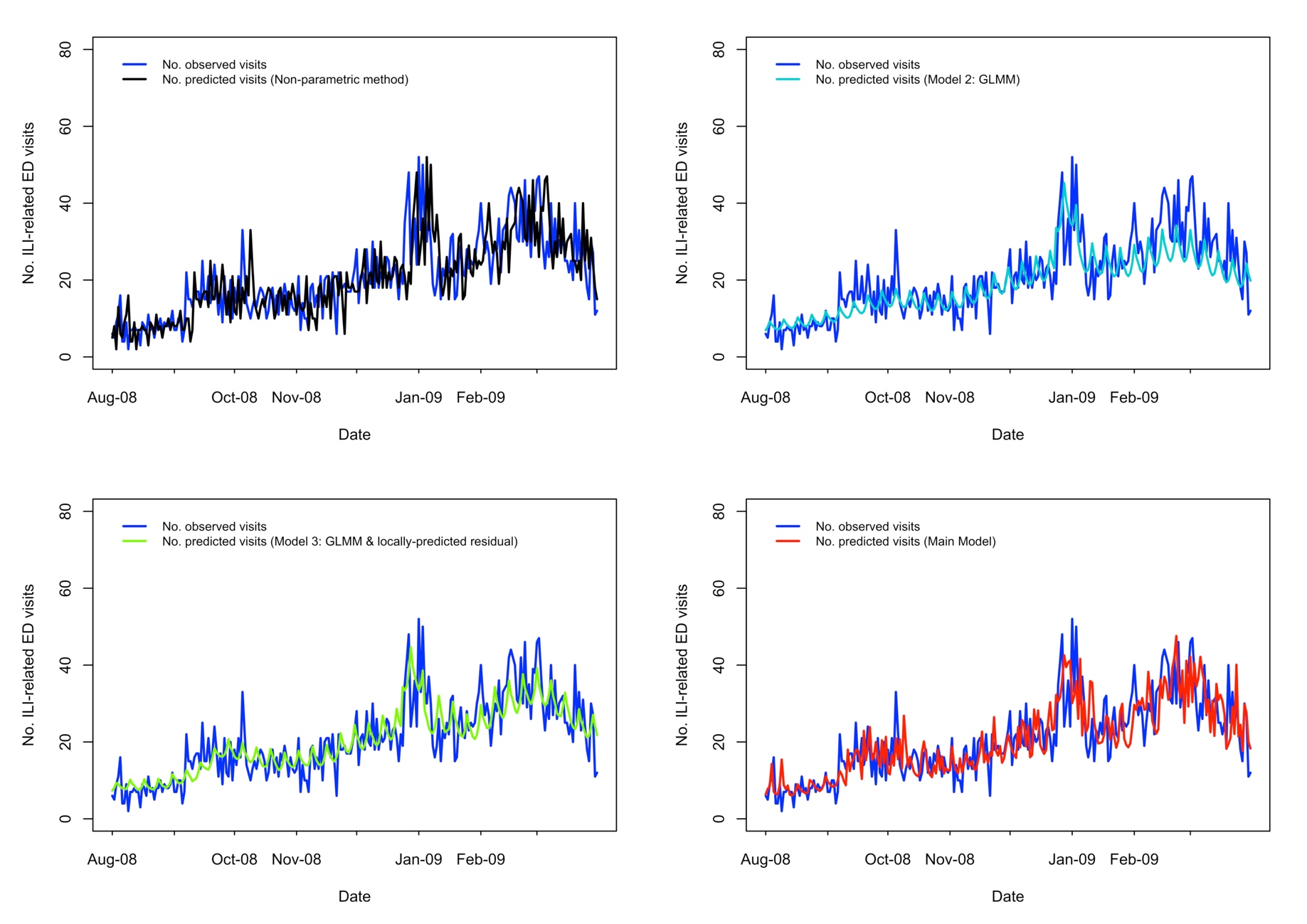
**

**Supplementary Figure S1.** Comparing predicted and observed number of influenza-like illness-related emergency department visits for each method for the pre-H1N1 period (August 1, 2008-March 31, 2009), Edmonton, Alberta.

**
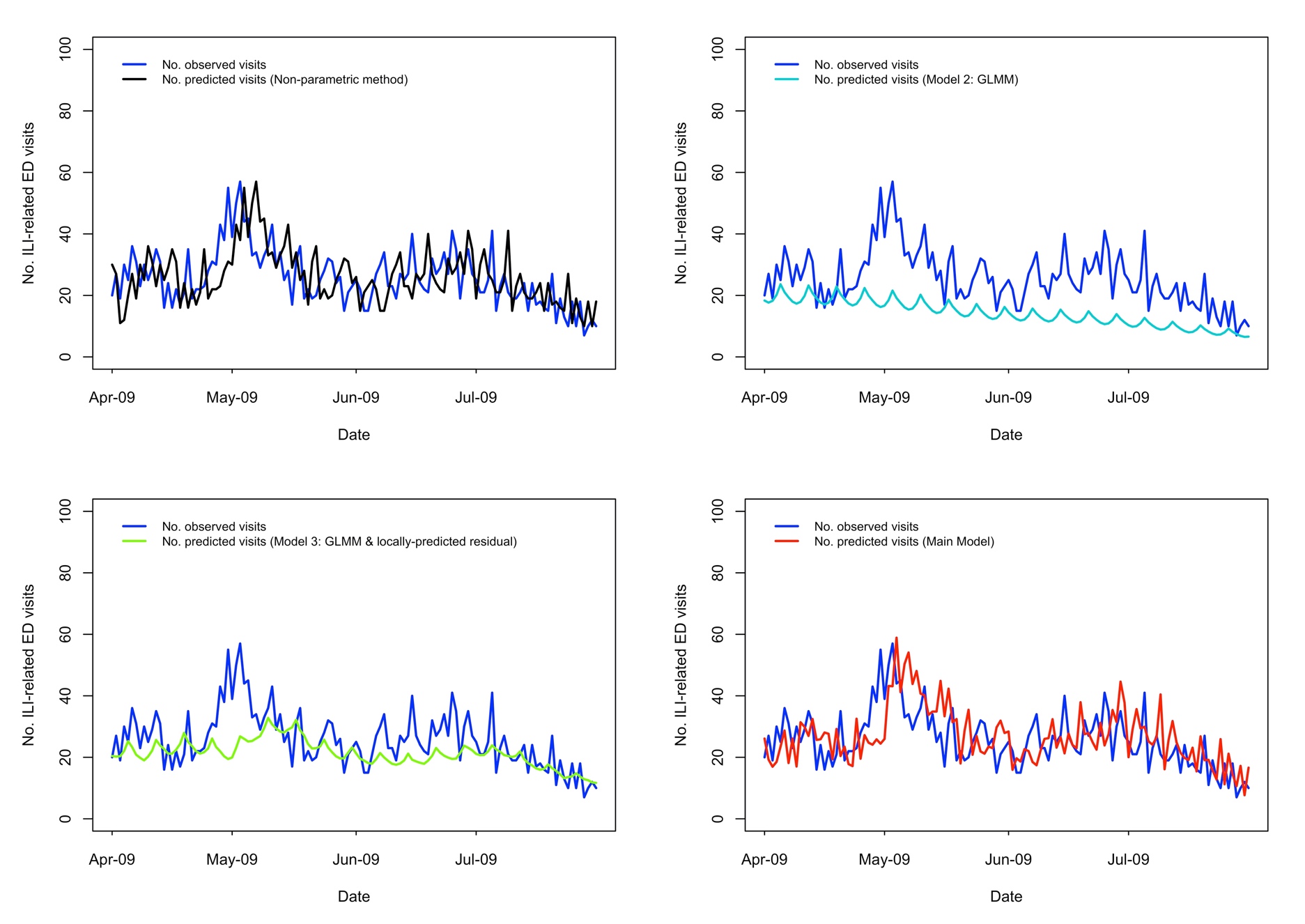
**

**Supplementary Figure S2.** Comparing predicted and observed number of influenza-like illness-related emergency department visits for each method for the first wave of the H1N1 pandemic (April 1-July 31, 2009), Edmonton, Alberta.


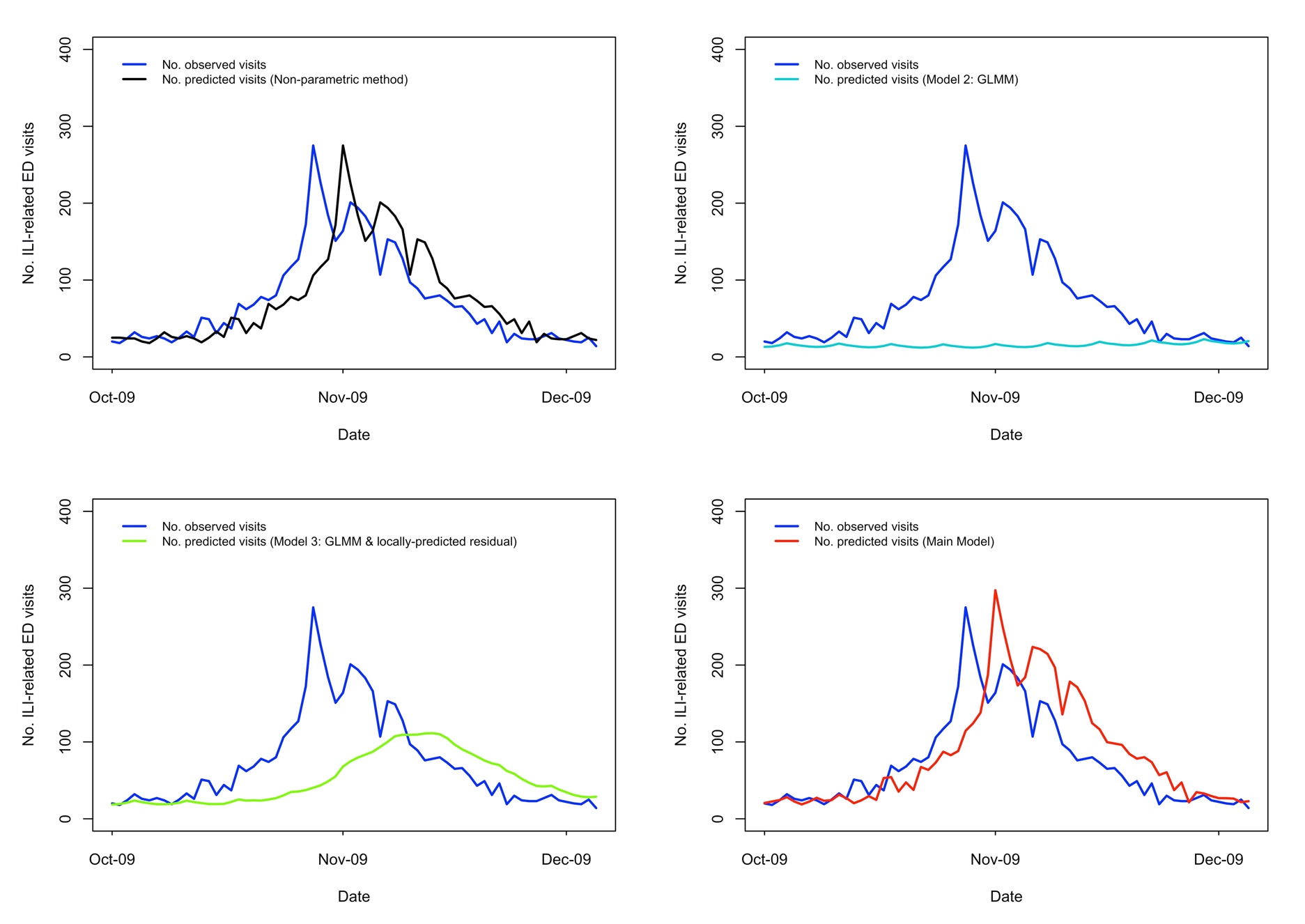


**Supplementary Figure S3.** Comparing predicted and observed number of influenza-like illness-related emergency department visits for each method for the second wave of the H1N1 pandemic (October 1, 2009-December 5, 2009), Edmonton, Alberta.

**
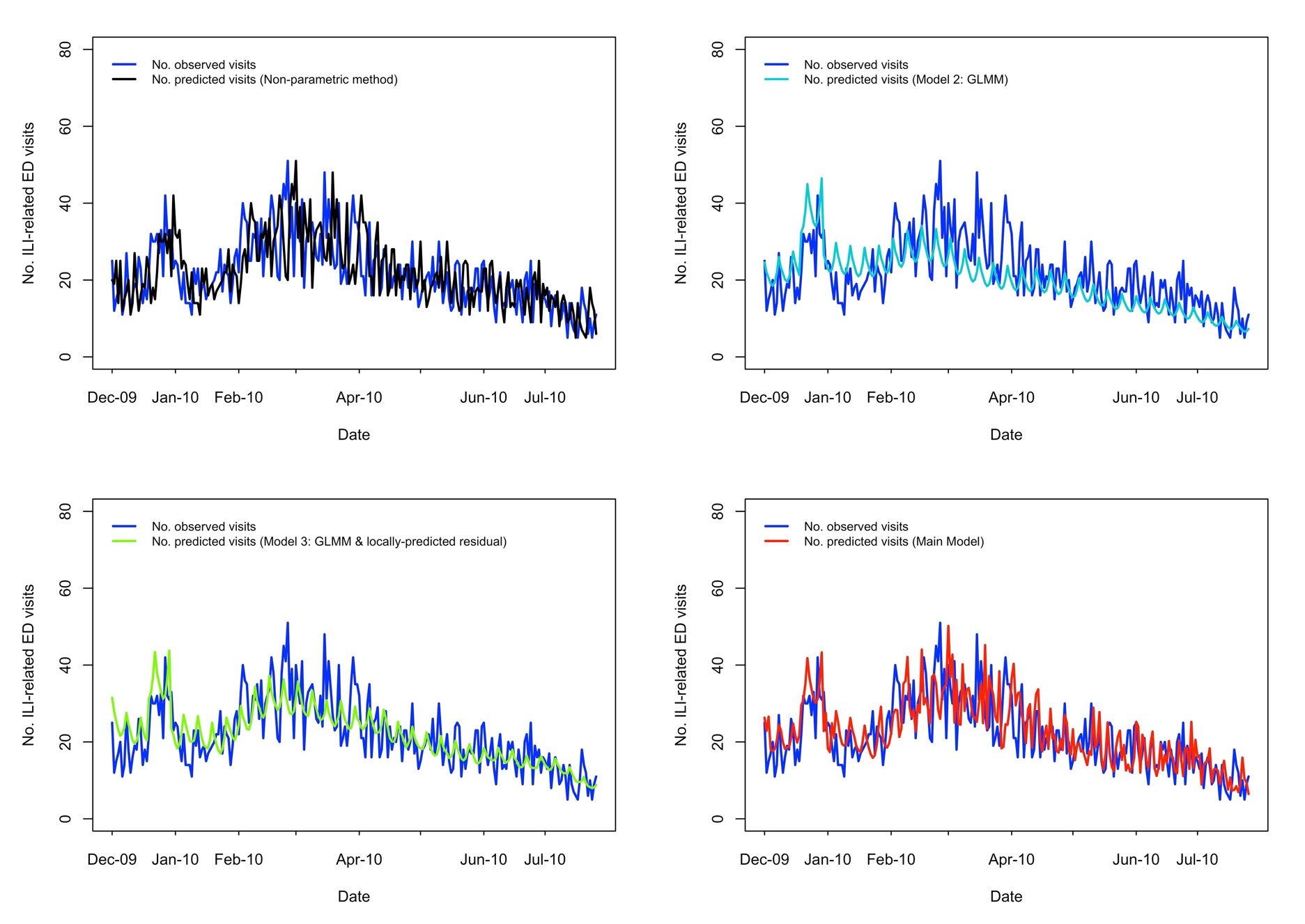
**

**Supplementary Figure S4.** Comparing predicted and observed number of influenza-like illness-related emergency department visits for each method for the post-H1N1 period (December 6, 2009-July 31, 2010), Edmonton, Alberta.


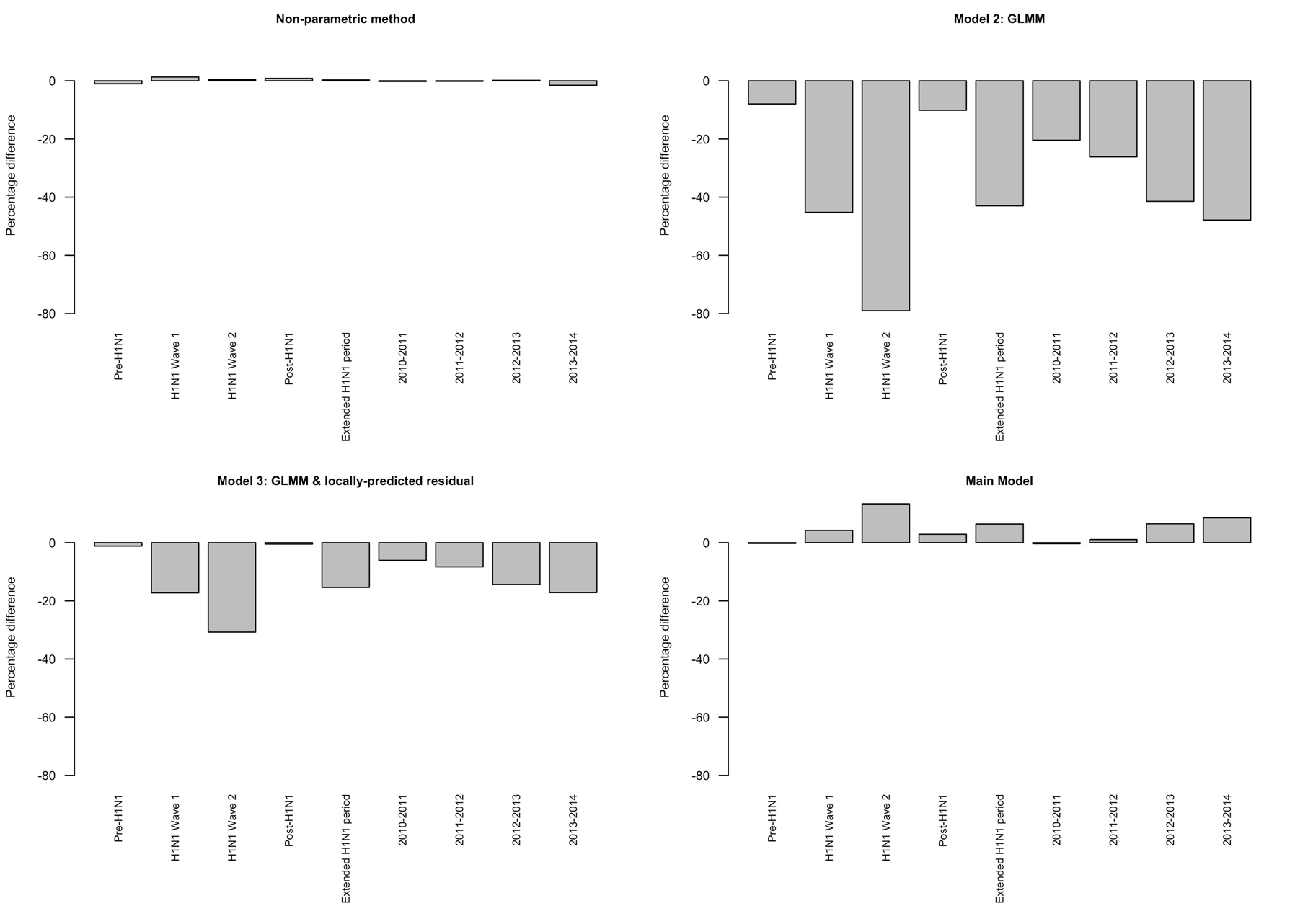


**Supplementary Figure S5.** Relative percentage difference between the number of influenza-like illness-related emergency department visits predicted by each model and number of visits observed, by period. Note: Relative percentage difference = (predicted volume - observed volume)/observed volume x 100


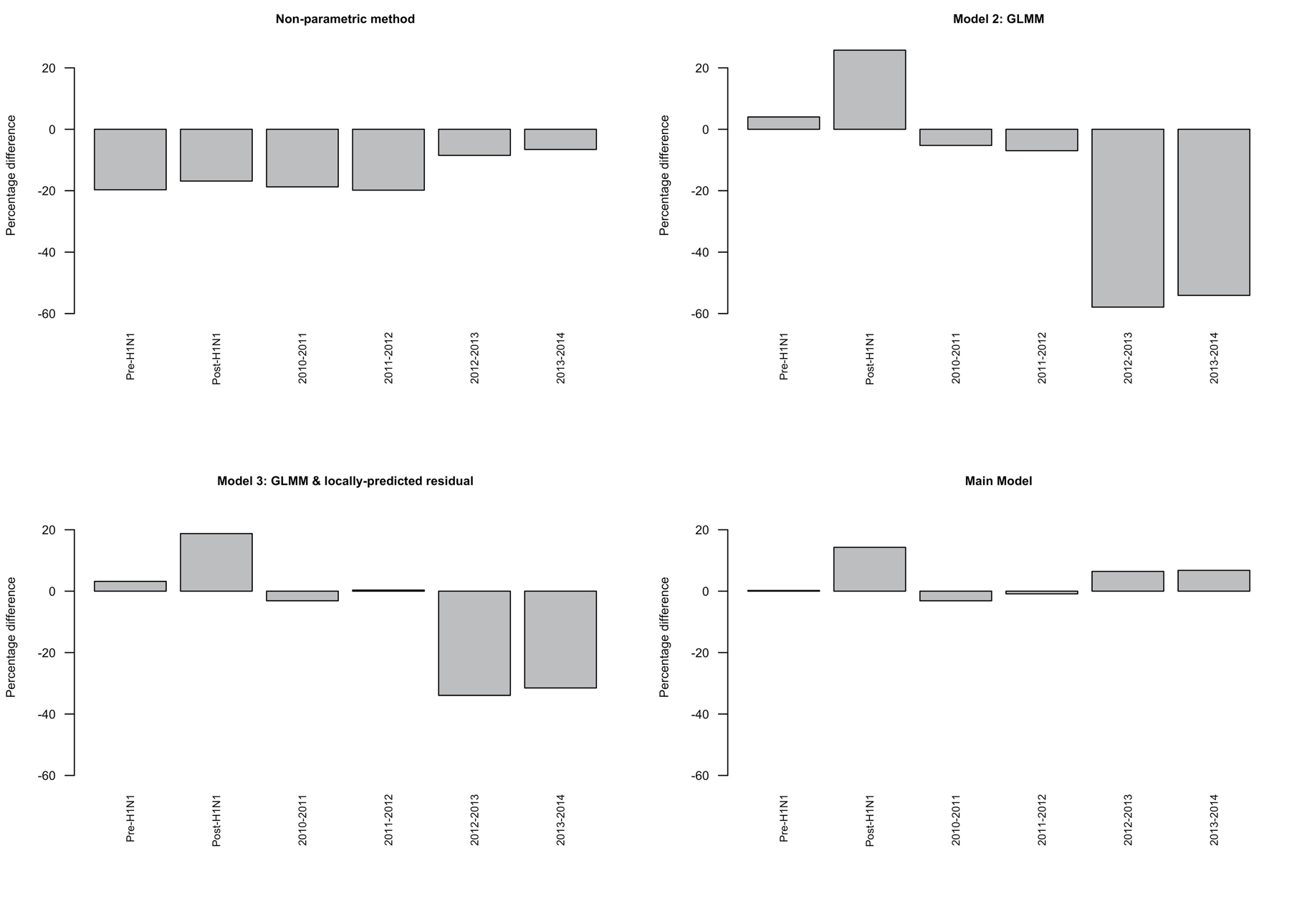


**Supplementary Figure S6.** Relative percentage difference between the number of influenza-like illness-related emergency department visits predicted by each model and number of visits observed, by period, limited to the Christmas-New Year holidays. Note: Relative percentage difference = (predicted volume - observed volume)/observed volume x 100 and is calculated for the entire period

**Notes on the Supplementary Figures S5 and S6**

Supplementary Figure S5 shows that the ILI-related ED visit volumes estimated by the Non-Parametric Model were closest to the observed volumes; however, this is expected because these estimates are the observed values offset by 3 days. Aside from this approach, the Main Model estimated ILI-related visit volumes that were closest to the observed volume in most periods. The exception was the Post-H1N1 period, when Model 3 was closer to the observed volume. The Main Model usually erred on the side of over- rather than under-estimation. In contrast, Model 3 and especially Model 2 underestimate the observed volume.

Supplementary Figure S6 shows that the ILI-related ED visit volumes estimated by the Main Model were closest to the observed volumes during the Christmas-New Year holiday periods for most periods: Pre-H1N1, Post-H1N1, and the final two seasons (2012-2013 and 2013-2014). In 2010-2011, the Main Model and Model 3 were the same. In 2011-2012, Model 3 was slightly better than the Main Model (0.35% vs. -0.87%).
